# Histone H3K4 Methyltransferases as Targets for Drug-Resistant Cancers

**DOI:** 10.3390/biology10070581

**Published:** 2021-06-25

**Authors:** Liu Yang, Mingli Jin, Kwang Won Jeong

**Affiliations:** 1 Collaborative Innovation Center for Chinese Medicine and Respiratory Diseases Co-Constructed by Henan Province & Education Ministry of P.R. China, Henan University of Chinese Medicine, Zhengzhou 450046, China; 15143159305@163.com; 2 Gachon Research Institute of Pharmaceutical Sciences, College of Pharmacy, Gachon University, 191 Hambakmoero, Yeonsu-gu, Incheon 21936, Korea; kimml1217@gachon.ac.kr

**Keywords:** histone H3K4, methyltransferase, resistant cancer, MLLs, inhibitors

## Abstract

**Simple Summary:**

Epigenetic modifications can regulate gene expression by altering chromatin structure. Since the beginning of comprehensive research into changes in gene expression due to early DNA methylation and histone acetylation, numerous experimental studies have revealed that the regulation of gene expression by histone methyltransferases plays an important role in cancer development, metastasis, and drug resistance. The enzyme responsible for H3K4 methylation, which is highly correlated with active transcription, has been studied in detail via impaired regulation of gene expression, following rearrangement of the mixed-lineage leukemia 1 (MLL1) gene. Other H3K4 methyltransferases have also been identified and have been shown to play a role in various cancers. In this review, we have examined the overall role of histone H3K4 methyltransferase in the development and progression of various cancers and its specific role in the development of drug-resistant cancers commonly encountered during chemotherapy. Additionally, we have discussed the H3K4-specific methyltransferase inhibitors currently under development for cancer treatment as well as their mechanisms of action.

**Abstract:**

The KMT2 (MLL) family of proteins, including the major histone H3K4 methyltransferase found in mammals, exists as large complexes with common subunit proteins and exhibits enzymatic activity. SMYD, another H3K4 methyltransferase, and SET7/9 proteins catalyze the methylation of several non-histone targets, in addition to histone H3K4 residues. Despite these structural and functional commonalities, H3K4 methyltransferase proteins have specificity for their target genes and play a role in the development of various cancers as well as in drug resistance. In this review, we examine the overall role of histone H3K4 methyltransferase in the development of various cancers and in the progression of drug resistance. Compounds that inhibit protein–protein interactions between KMT2 family proteins and their common subunits or the activity of SMYD and SET7/9 are continuously being developed for the treatment of acute leukemia, triple-negative breast cancer, and castration-resistant prostate cancer. These H3K4 methyltransferase inhibitors, either alone or in combination with other drugs, are expected to play a role in overcoming drug resistance in leukemia and various solid cancers.

## 1. Introduction

Epigenetics refers to heritable alterations in gene expression that are not caused by changes in the DNA sequence [1]. The main epigenetic modifications include DNA methylation, histone modification, miRNA-mediated modulation, and modulation of gene expression by alteration of chromatin structure [2,3]. Histone modification is classified into histone methylation, acetylation, ubiquitination, phosphorylation, and sumoylation [4,5]. Early epigenetic studies focused on changes in gene expression resulting from DNA methylation and histone acetylation. Extensive research has also been conducted on histone methylation, revealing that the regulation of bilateral gene expression by histone methylation plays an important role in cancer development, metastasis, and drug resistance [6,7,8,9,10]. 

Histone methylation, first discovered in the 1960s, occurs primarily at the arginine (R) and lysine (K) residues located in the histone H3, H4, and H2A tails [11,12]. Arginine residues can be mono- or di-methylated symmetrically or asymmetrically at the amine group sites of the H3R17, H3R26, H3R2, H4R3, H2AR3, and H3R8 residues [13,14,15,16,17,18,19]. Lysine residues can be modified by mono-, di-, or tri-methylation of H3K4, H3K9, H3K27, H3K36, H3K79, and H4K20 residues [20,21,22,23,24,25,26]. Histone methylation is controlled by site-specific histone methyltransferases (HMTs) and can be reversed by specific demethylases. Numerous experimental studies have suggested that the regulation of gene expression by HMTs and demethylases is closely associated with cancer development [27,28,29].

Resistance to anticancer drugs remains a significant challenge in tumor treatment and recent studies have recommended targeting HMTs and demethylases to counter drug resistance. In this review, we examine the overall role of histone H3K4-specific methyltransferase in cancer and specifically explore the role of HMT in drug-resistant cancers. H3K4 methyltransferase inhibitors are currently being developed for cancer treatment, and their mechanisms of action are also discussed in this review. The HMTs and inhibitors discussed in this review are cited from primary literature. Data on inhibitors used in clinical studies were retrieved from ClinicalTrials.gov, a database which is maintained by the U.S. National Library of Medicine.

## 2. Classification of H3K4 Methyltransferases

### 2.1. KMT2/MLL Family

The major histone H3K4 methyltransferase in mammals is in the KMT2 (MLL) family, which has six members (KMT2A-D, KMT2F, and KMT2G) [30]. The mixed-lineage leukemia (MLL) protein has been studied in detail due to the dysregulation of gene expression caused by the rearrangement of the MLL1 (KMT2A) gene in acute leukemia [31,32]. Binding of DOT1L (KMT4) or the super elongation complex (SEC) occurs via a fusion partner (AF4, AF9, AF10, ELL, or ENL) newly bound to the N-terminus of the MLL1 protein, resulting in the introduction of the H3K79me mark at the specific site in the target gene (e.g., *HOXA9* or *MEIS1*) [33]. In contrast, the wild-type KMT2 protein family exists as large complexes with common interacting subunit proteins (WRADs) such as WD-repeat protein 5 (WDR5), retinal blastoma binding protein 5 (RBBP5), ASH2L, and DPY30 [22,34,35,36]. The SET [Su(var)3-9, enhancer-of-zeste, and trithorax] domains exert major enzyme activities against KMT2 proteins. Additionally, binding to WDR5 via a conserved arginine-containing WDR5 interaction (Win) motif located at the N-terminus of the SET domain of the MLL family is essential for the assembly and enzymatic activity of the MLL core complex [37,38]. Additional biochemical experiments have also shown that the stable heterodimer formed by ASH2L and RbBP5 promotes the interaction between the SET domain and the substrate, thereby indicating increased overall methyltransferase activity of the KMT2 protein complex which is attributable to the interaction [39]. 

**Figure 1:**
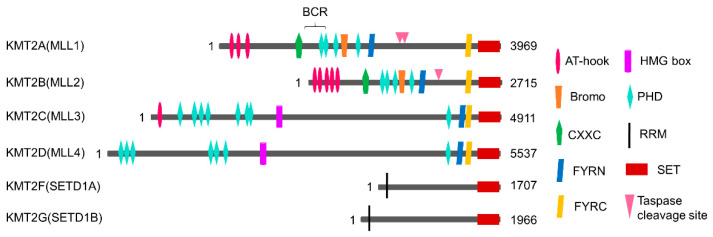
Structures of the members of the KMT2/MLL1 protein family. The numbers represent amino acids in each protein. AT-hook, adenosine-thymidine-hook; Bromo, Bromodomain; CXXC, Zinc-finger-CXXC domain; FYRN, Phe/Tyr-rich N-terminal domain; FYRC, Phe/Tyr-rich C-terminal domain; HMG, high mobility group; PHD, plant homeodomain; RRM, RNA recognition motif; SET, Su(var)3-9, Enhancer-of-zeste and trithorax.; BRC, breakpoint common region. Adapted from [44].

The KMT2 family can be divided into the following three subgroups based on the type of domain it contains: KMT2A and KMT2B (MLL1 and MLL2), KMT2C and KMT2D (MLL3 and MLL4), and KMT2F and KMT2G (SETD1A and SETD1B, Figure 1). KMT2A and KMT2B have taspase 1 cleavage sites. The two protein fragments are produced as a result of taspase 1-induced cleavage and are linked by FY-rich N-terminal (FYRN) and FY-rich C-terminal (FYRC) domains to form a functional heterodimeric complex [40]. MLL1–4 contain the plant homeodomain (PHD), an epigenetic reader of H3K4me3 [41]. Additionally, the CXXC zinc-finger domain is present in MLL1 and MLL2 and the AT-hook region of MLL1–3, and the high mobility group (HMG) box of MLL3 and MLL4 is associated with DNA binding [42]. The SETD1A and SETD1B proteins characteristically contain an RNA recognition motif (RRM) [43]. 

Despite structural similarities due to shared key subunits, members of the KMT2 protein family have specific enzymatic reactions. In vitro studies have shown that the MLL1 and MLL2 core complexes have mono-, di-, and low tri-methylation activity on H3K4 [45,46]. The MLL3 and MLL4 core complexes exhibit mainly mono-methyl transfer activity [47,48]. In contrast, the SETD1A and SETD1B core complexes can perform all mono-, di-, and tri-methylation processes on H3K4 [48,49]. However, in cells, including mouse embryonic fibroblasts (MEFs) and mouse embryonic stem (ES) cells, MLL1 and MLL2 have been shown to primarily implement H3K4me3 at gene promoters [50,51]. MLL3 and MLL4 are responsible for the bulk of mono- and di-methylation of H3K4 in the enhancer region [47,52]. It has been suggested that SETD1A and SETD1B contribute mainly to di- and tri-methylation of H3K4 in cells [53,54]. These enzymatic properties in cells can also be confirmed by tracking the location of the KMT2 protein complex distributed throughout the genome. Consistent with the selective methylation properties of KMT2 enzymes at the cellular level, a series of chromatin immunoprecipitation sequencing (ChIP-seq) experiments demonstrated that MLL1 and MLL2 were recruited to both gene promoters and enhancers [55,56], while MLL3 and MLL4 were highly enriched in enhancers [55]. Furthermore, SETD1A and SETD1B were generally found to be bound near transcription start sites (TSSs) [57].

In addition to the shared common core subunits, the proteins in the KMT2 family also have several unique complex-specific subunits. These structural features provide the KMT2 complex with functional diversity for recruitment to the chromatin, in addition to enzymatic activity. For example, MLL1 and menin form an interaction surface for lens epithelial-derived growth factor (LEDGF), which can bind to chromatin via the PWWP domain [58,59,60]. The additional subunits associated with the MLL3 and MLL4 complexes are PTIP (PAX transactivation domain-interacting protein) and PA1 (PTIP-associated 1), which have robust HMT activity on histone H3K4 [61]. An extensive interactome study also revealed that CXXC-finger protein 1 (CFP1) and WD-repeat domain 82 (WDR82) interact only with SETD1A and SETD1B [40].

### 2.2. SMYD Family

Among the SET (suppressor of variegation, enhancer of zeste, trithorax) and MYND (myeloid-nervy-DEAF1) family proteins (SMYD1-5), SMYD1, SMYD2, and SMYD3 have methyltransferase activity specific to histone H3K4 [62,63,64]. These three members have a common tetratricopeptide (TPR)-like domain, which is an important motif for protein–protein interactions, at their C-termini [62,65,66]. The split-SET (N-SET and C-SET) domains and the MYND domains located between them form their characteristic active enzyme sites; particularly, the intermediate SET spacer (I-SET) located at the C-terminus of MYND is a linker region that plays an important role in regulating substrate selectivity [66]. 

SMYD1 was initially shown to regulate cardiac differentiation and morphogenesis by binding to skeletal NACA (skNAC), a cardiac and muscle-specific transcription factor [67,68]. The methyltransferase activity of SMYD1, which targets histone H3 lysine4, was first identified in 2006, and the important role of SMYD1 in muscle fiber maturation has since been elucidated [69]. SMYD1 mediates di- and tri-methylation of H3K4 residues [70]. SMYD2 was first identified as a methylation enzyme of histone H3K36 [71]; subsequently, proteomic and genomic studies have demonstrated its ability to catalyze the methylation of H3 lysine 4 and several non-histone targets [63]. HSP90α regulates the selective enzymatic reaction between SMYD2 and two different substrates (H3K36 and H3K4). In the presence of HSP90α, SMYD2 binds to HSP90α, enhancing its methyltransferase activity and specificity towards H3K4 [63]. In the absence of HSP90α, SMYD2 acts as a H3K36 methyltransferase [63,71]. SMYD2 also acts on non-histone proteins. When lysine 370 of p53 is mono-methylated by SMYD2, p53-mediated transcriptional regulation is inhibited [72,73]. The retinoblastoma tumor suppressor gene, *RB,* is also a substrate for SMYD2, and the methylation of the lysine 860 residue facilitates the binding of *RB* to the transcriptional repressor L3MBTL histone methyl-lysine binding protein 1 (L3MBTL1) [74]. SMYD3 plays a pivotal role in human carcinogenesis and methylates H3K4, H4K5, and H4K20, which are involved in gene regulation [66,75]. Additionally, it has recently been reported that SMYD3 can methylate vascular endothelial growth factor receptor 1 (VEGFR1) at lysine 831, thus enhancing VEGFR1′s kinase activity in HEK293 cells [76].

### 2.3. Other H3K4 Methyltransferases

In addition to the two main H3K4 methyltransferase families discussed above, other lysine methyltransferases can also methylate H3K4. SET7/9 (KMT7) is a SET domain-containing lysine methyltransferase that has been shown to monomethylate H3K4 in vitro and in vivo and activates gene expression [77,78,79]. SET7/9 monomethylates various non-histone proteins, including p53 [80], DNA cytosine methyltransferase 1 (DNMT1) [81], E2 promoter-binding factor 1 (E2F1) [82], hypoxia-inducible factor 1α (HIF1α) [83], and Yes-associated protein (YAP) [84], which participate in a series of cellular processes. PRDM9 (KMT8B/MEISETZ), a member of the PRDI-BF1 and RIZ homology domain-containing family (PRDM), has also been shown to methylate H3K4 and H3K36 [85,86,87]. PRDM9 contains an N-terminal KRAB (Krüppel-associated box)-related domain for protein–protein interaction, an SSX repression domain (SSXRD) involved in nuclear localization, a canonical SET domain, and a C-terminal tandem array of C2H2 zinc-finger motifs with sequence-specific DNA binding activity [88]. 

### 2.4. Distribution of H3K4 Methylation and H3K4 Readers

Although all types of H3K4 methylations have a strong correlation with active transcription [22], the distribution of H3K4 methylation types on chromosomes is distinctly different [89,90,91,92,93,94]. For example, H3K4me1 is rich in active and priming enhancer regions. H3K4me2 is also an enhancer marker, although predominantly toward the 5′ end of transcribing genes. In contrast, H3K4me3 is considered a hallmark of promoters and is predominantly localized near TSSs where it is responsible for RNA pol II recruitment for the activation of gene expression. 

Each state of H3K4 methylation must be recognized by other cellular proteins known as “readers” to recruit distinct downstream effectors and regulate gene expression [95]. Numerous chromatin modifiers have specialized domains that allow them to act as H3K4 methylation readers. Most methylated H3K4 reader proteins contain a plant homeodomain (PHD), and some chromodomains and Tudor domains also appear to bind to H3K4me. For example, BPTF (bromodomain and PHD finger transcription factor), which is a subunit of the ATP-dependent chromatin-remodeling complex nucleosome remodeling factor (NURF), is preferentially associated with H3K4me3 tails [96]. The TAF3 subunit of the TFIID transcription factor also recognizes H3K4me3, directing TFIID recruitment [97]. CxxC-finger protein 1 (CFP1), the histone demethylase plant homeodomain finger 2 (PHF2), spindlin1 (SPIN1), and NUP98 fusion partners plant homeodomain finger 23 (PHF23) are also specifically recognized and interact with H3K4me3 via the PHD domain, thus participating in gene expression [98,99,100,101]. pygopus family PHD finger 2 (PYGO2) has been reported to bind di- and tri-methylated lysine 4 of H3 [102]. CHD1, an ATP-dependent chromatin-remodeling enzyme, recognizes H3K4me2 and H3K4me3 through its two N-terminal chromodomains [103]. The chromo-barrel domain of Tip60 acetyltransferase is recruited to the enhancer region by binding to H3K4me1, thus inducing estrogen-induced transcription [104]. Researchers recognize that the H3K4me mark sometimes invokes the binding of other executor proteins. For example, Sgf29 selectively interacts with H3K4me2/3 marks via the Tudor domain to recruit the SAGA complex and mediate histone H3 acetylation [105]. Additionally, ING4, a subunit of the HBO1 histone acetyltransferase (HAT) complex, recognizes H3K4me3 via the PHD domain, enhancing HBO1 acetylation activity and resulting in increased H3 acetylation in the ING4 target promoter [106].

## 3. H3K4 Methyltransferases in Drug-Resistant Cancers

Controlling epigenetic factors, including histone methyltransferases and demethylases, has recently emerged as a way of overcoming drug resistance, which is often encountered in chemotherapy [7,107]. In the next section, we investigate the function of H3K4 methyltransferase in cancer, specifically drug-resistant cancer.

### 3.1. H3K4 Methyltransferase in Breast Cancer

Extensive evidence supports the crucial role of H3K4 methyltransferase in breast cancer development. MLL2 interacts with estrogen receptor α (ERα) and regulates ERα target gene expression to mediate breast cancer growth [108]. Moreover, MLL2 levels are elevated in cell lines and in invasive carcinomas in breast and colon cancer patients [109]. MLL3, one of the most commonly mutated proteins in breast cancer, functions as another major regulatory factor for ERα expression [110]. Recent studies have shown that enhanced MLL3 and SETD1A expression promotes ERα expression to support tamoxifen-resistant breast cancer proliferation, and genome-wide histone methylation studies have revealed that MLL3 is required for acetylation of H3K27 and monomethylation of H3K4 in the ERα enhancer [111]. Similarly, depletion of MLL4 decreased H3K4me3 levels and increased H3K27me3 levels in *MMP9*, *MMP11*, and *SIX1* genes in MDA-MD-231 cells [112]. These results suggest that H3K4 methyltransferase may link H3K4 methylation and H3K27 acetylation via a specific mechanism in breast cancer cells, similar to ES cells [112,113,114]. This finding was explained by a study of MLL4 expression in breast cancer cells. MLL4 coupled with histone H3 lysine 27 (H3K27) demethylase UTX (KDM6A) showed coordinated regulation of breast cancer proliferation and invasion [112]. Thereafter, studies revealed that the UTX-MLL4 complex remarkably increased binding of H3K27 acetyltransferase p300 to the target chromatin region, further enhancing H3K27 acetylation and resulting in increased enhancer activity for gene activation [115]. The relationship between H3K4 methylation and H3K27 acetylation has been demonstrated in various cell differentiation processes such as adipogenesis, myogenesis, and ESC differentiation [52,116,117].

The frequent duplication or overexpression of MLL1 in breast cancer cells shows its potential as a target molecule for breast cancer treatment [118]. In MCF-7 cells, MLL1 and SETD1A have been reported to be responsible for H3K4 methylation during estrogen-induced transcription of ER target genes [104]. One study also showed that SETD1A regulated breast cancer metastasis by activating MMP expression [113], while another reported that the amplification of SETD1A in mixed ductal and lobular breast cancer (MDLC) regulated the mitotic process by increasing the H3K4me3 marker in the promoter region of mitosis-and-DNA-damage response genes. Additionally, SETD1A regulates the expression of several genes that orchestrate mitosis, the cell cycle, and DNA damage responses through H3K4 methylation of promoters in breast and lung cancer cells. Depletion of SETD1A induces the tumor-suppressing effect of senescence; therefore, SETD1A appears to be essential for maintaining mitosis and proliferation of cancer cells [114]. We recently examined the function of SETD1A in tamoxifen-resistant breast cancer [119]. SETD1A activated a subset of ER-positive target genes by increasing H3K4 methylation and accessibility to the chromatin region of the ERα target gene in ER-positive breast cancer cells, supporting ERα recruitment. Additionally, SETD1A induced the proliferation and migration of ER-positive breast cancer cells by regulating the expression of survival- and migration-related genes in an ER-independent manner. These results suggest that SETD1A may play an important role in acquiring resistance to hormone therapy drugs in breast cancer, independent of ER signaling [119]. Notably, the intracellular protein levels of SETD1A are upregulated in other breast cancer subtypes, including ER-positive, HER2-positive, and TNBC breast cancer compared with normal breast cancer cells, and miR-1915-3p regulates this process. We also found an overlap between genes regulated by SETD1A and tamoxifen resistance-specific genes in ER-positive breast cancer cells, suggesting that SETD1A may be involved in tamoxifen resistance. Indeed, in tamoxifen-resistant breast cancer, SETD1A silencing dramatically reduces the expression of cell growth genes (e.g., *EGFR* and *MYC*) required for the development of tamoxifen resistance, and inhibits cell proliferation by inducing cell cycle arrest [119]. Interestingly, SETD1B, which is structurally similar to SETD1A, plays an important role in the survival and pathogenesis of TNBC, regardless of H3K4 methyltransferase activity, e.g., by forming COMPASS complexes in the cytoplasm and regulating ADIPOR1 signaling by interacting with BOD1 [116].

SMYD2 overexpression promotes the progression of triple-negative breast cancer and is clinically associated with poor prognosis [117]. The ability of SMYD3 to increase the expression of the oncogene WNT10B and promote the epithelial–mesenchymal transition (EMT) process is thought to contribute to metastatic breast cancer [120,121]. A previous study on breast cancer reported that excessive expression of SMYD3 increased cell tolerance to cisplatin treatment in MCF-7 cells [122]. Additionally, SMYD3 depletion, in conjunction with cisplatin treatment, inhibited cell viability and mitochondrial membrane potential. These data suggest that SMYD3 is a key factor in determining cisplatin sensitivity and plays an important role in cisplatin resistance in cancer.

In addition to regulating the expression of RUNX2 and some redox enzymes to maintain redox homeostasis [123,124], SET7/9 promotes breast cancer development by stabilizing the ER through methylation of the ER K302 residue, aiding in efficient recruitment to and transactivation of the target genes [125]. However, another study showed a negative feedback loop between SET7/9 and DNMT1. In contrast to DNMT, SET7/9 is significantly less expressed in TNBC and inhibits EMT when overexpressed in these cells. Moreover, loss of SET7/9 increases breast cancer stem cell-like properties and enhances the EMT process, which is relevant to tumor resistance, suggesting that SET7/9 acts as a suppressor in breast cancer [126]. This function of SET7/9 is caused by the negative regulation of stability by methylation of DNMT and E2F1. These results suggest that SET7/9 could be used as a biomarker to predict the potential for metastasis and resistance to anti-estrogen therapy in breast cancer patients.

### 3.2. H3K4 Methyltransferase in Colorectal Cancer

Previous reports have shown that the restoration of MLL3 expression enhances the H3K4me1 profile and represses colorectal cancer (CRC) growth, suggesting that MLL3 functions as a tumor suppressor [127]. MLL3 and MLL4 are coactivators of p53 and act as tumor suppressors by labeling H3K4me3 to express endogenous p53-target genes induced by DNA-damaging agents, such as doxorubicin [128]. Additionally, SET7/9 has a tumor suppressor function which involves it interacting with HDAC6 and suppressing HDAC6-mediated activation of the ERK signaling pathway in colon cancer [129]. In contrast, MLL4 directly interacts with mutated p53 (p53^R273H,P309S^) to increase H3K4me1 and histone H3 lysine 27 acetylation (H3K27ac) levels, thus activating the chronic TNFα signaling pathway which accelerates colon cancer invasion [130]. In colorectal cancer, SETD1A has been reported to interact with β-catenin to regulate Wnt signaling and promote cancer cell growth [131]. When the YAP protein, a SETD1A non-histone substrate, is methylated by SETD1A, its migration out of the nucleus is inhibited and, consequently, YAP-TEAD transcriptional activity is increased, leading to colon cancer cell proliferation and tumor formation [132]. Furthermore, SETD1B frameshift mutations play an important role in the tumorigenesis of colorectal and gastric cancers with high microsatellite instability [133]. Increased levels of SMYD2 upregulate MDR1/P-glycoprotein expression via the MEK/ERK/AP-1 pathway in colon cancer, which promotes oxaliplatin resistance in colon cancer [134]. SMYD3 interacts with the RNA helicase HELZ, forming a complex with RNA polymerase II and promoting cancer cell proliferation in colorectal and hepatocellular carcinomas [64]. 

### 3.3. H3K4 Methyltransferase in Prostate Cancer

A limited number of H3K4 methyltransferases have been studied in prostate cancer. The MLL1 complex binds directly to the androgen receptor (AR) through a menin subunit, acting as a co-activator of AR signaling. Additionally, menin expression is upregulated in castration-resistant prostate cancer and is correlated with low overall survival in individuals diagnosed with prostate cancer [135]. MLL2 is also considered a potential therapeutic target because it activates the PI3K/EMT process [136] and induces DNA damage in prostate cancer [137]. SMYD3 depletion inhibits prostate cancer progression through a mechanism that blocks the transcription of AR or cyclin D2 [138,139]. SET7/9 interacts directly with AR and enhances AR transcriptional activity by methylating the K632 residue of AR. It not only plays a proliferative role in prostate cancer but is also involved in TNFR and PTEN/PI3K/AKT signaling [140,141]. Whole transcriptome sequencing revealed that SET7/9 has a frameshift mutation that leads to changes in chromatin accessibility during co-transcriptional RNA processing in castration-resistant prostate cancer [142].

A study conducted by our group has recently revealed that SETD1A, which is present at higher levels in metastatic castrate-resistant prostate cancer (mCRPC) than in primary prostate cancer cells, contributes to the tri-methylation of H3K4 by binding to E2F1 in the promoter region of the *FOXM1* gene, a cancer cell proliferation-specific transcription factor. SETD1A is also essential for the expression of stem cell factors, e.g., octamer-binding transcription factor 4 (OCT4) and plays an important role in the proliferation of prostate cancer stem cells, which is important in metastatic CRPC tumor formation. Particularly, the expression of SETD1A is significantly correlated with the survival rate of prostate cancer patients, and the high expression of the SETD1A-FOXM1 pair is highly correlated with a poor prognosis, suggesting that it can be used as an important marker for predicting the proliferation and prognosis of mCRPC [143]. 

### 3.4. H3K4 Methyltransferase in Leukemia

MLL1 was initially associated with acute myeloid and lymphoblastic leukemia (AML, ALL) through chromosomal rearrangement [144,145]. MLL1 rearrangements on chromosome 11q23 result in a chimeric gene consisting of the N-terminus of MLL1 fused to the C-terminus of fusion partners. Four frequent fusion partners, AF4, AF9, AF10, and ENL, accounted for more than 70% of all observed rearrangements in patients. Importantly, MLL1-rearranged leukemia predicts a poor prognosis in patients [146]. Biological studies have suggested a major mechanism by which MLL1 fusion proteins drive leukemogenic gene expression dependent on its binding with chromatin-associated protein complexes, including menin, SEC, and DOT1L (disruptor of telomeric silencing 1-like). Binding of menin to the MLL1 portion of the fusion protein is critical for MLL1 fusion-mediated transformation [147]. Interaction with SEC is involved in the activation of RNA Pol II for transcriptional elongation [148]. DOT1L, which binds to the fusion partner site of the rearrangement protein, is a histone H3 lysine 79 (H3K79) methyltransferase, wherein abnormal methylation occurs at the H3K79 site at the MLL1 fusion target gene sites, leading to abnormal expression of genes responsible for cell proliferation [149,150]. 

Apart from MLL1-rearranged leukemias, there are reports on the function of wild-type H3K4 methyltransferase in leukemia. The wild-type MLL1 protein, along with the MLL1-AF9 fusion protein, participates in abnormal H3K4 and H3K79 methylation in the *HOX* gene region, causing leukemogenesis [151]. Additionally, SETD1A acts as an essential regulator of gene expression required for DNA damage reactions through interaction with cyclin K using the non-catalytic site, the FLOS domain. This SETD1A/cyclin K pathway supports MLL1-rearranged and non-MLL1-rearranged leukemia cell growth [152]. The loss of wt MLL2 significantly reduced MLL-AF9-transformed cell survival, and co-deletion with MLL1 further reduced leukemia cell proliferation by regulating major AML survival pathways [153]. However, MLL2 appears to have a completely different function in chronic myelogenous leukemia (CML) that is resistant to tyrosine kinase inhibitors (TKIs). The expression level of MLL2 decreased in (TKI)-resistant CML. Furthermore, when TKI-sensitive CML cells are treated with TKIs, such as dasatinib or nilotinib, MLL2 expression, which can increase p21 expression, is induced while the expression of CDK2, CDK4, and cyclin B1 is attenuated, resulting in a higher rate of cell death [154]. MLL3 is often deleted from AML, and its function as a tumor suppressor has been proposed; however, the exact mechanism is unknown [155].

SMYD2 overexpression promotes disease progression and is correlated with poorer outcomes in childhood acute lymphoblastic leukemia (ALL) [156]. Specifically, the level of SMYD protein in AML appears to correlate with the level of the SET7/9 protein [157]. When SMYD2 is downregulated, leukemia cells transition to a quiescent state after anti-leukemia chemotherapy treatment, resulting in increased resistance to anticancer drugs, which is thought to be due to increased SET7/9 expression in response to decreasing SMYD2 levels. In this case, a pharmacological SET7/9 inhibitor was effective at low-expressing SMYD2 AML. However, downregulation of SET7/9 through hypermethylation of the SET7/9 promoter was also observed in AML patient cells, and further studies are required to identify the target mechanism underlying the regulation of SMYD2 and SET7/9 [158].

There are no detailed mechanistic studies on the relationship between PRDM9 and cancer. Aberrant PRDM9 expression appears in 32 different cancer types and differential RPDM9 expression is linked to genomic instability and transcriptomic landscape in tumors [159]. Excess rare PRDM9 allelic forms were found in B-cell precursor acute lymphoblastic leukemia (B-ALL) in children, which might influence genomic instability leading to aneuploidies formation related to childhood leukemogenesis [160]. As we have seen so far, alterations in H3K4 methyltransferases either promoted or suppressed various cancers via multiple mechanisms which need to be explored further. 

### 3.5. H3K4 Methyltransferase in Gastric Cancer

In gastric cancer, the functions of H3K4 methyltransferases are contradictory depending on the type of protein. MLL2 is an oncogene that modulates proliferation and apoptosis in gastric cancer [161]. In contrast, restoring MLL3 repressed the proliferation of diffuse-type gastric adenocarcinoma (DGA) by targeting the EMT pathway [162]. Most recently, SETD1A overexpression was also found in HIF1α-related gastric cancer, enhancing glycolysis and promoting gastric cancer progression [163]. Similarly, SMYD2 is overexpressed in gastric cancer and promotes gastric cancer progression, which is associated with worse clinical outcomes [164]. SMYD3 promotes cell proliferation, migration, and invasion in gastric cancer by mediating the ATM-CHK2-p53/Cdc25C pathway [165,166]. In contrast, SET7/9 expression was downregulated in gastric cancer, thus it is considered a tumor suppressor, acting by either activating SREK1IP1 expression or inhibiting three MMP genes (*MMP1*, *MMP7*, and *MMP9*) [167].

### 3.6. H3K4 Methyltransferase in Other Cancers

Overexpression of P-glycoprotein (Pgp) encoded by human multidrug resistance 1 (MDR1) is closely linked with cancer drug resistance by serving as an ATP-dependent efflux pump which reduces the anticancer agents in resistant cells [168,169]. Recently, it was demonstrated that MLL1-mediated H3K4me3 can activate MDR1 transcription, and MLL1 depletion conferred cells sensitive to multiple chemotherapeutic agents such as paclitaxel, vinblastine, and daunorubicin [170]. In salivary gland squamous and head and neck carcinomas, MLL1 was shown to associate with Wnt/β-catenin signaling to regulate tumor initiation and proliferation [171,172]. In another study, the mRNA and protein levels of MLL1 were higher in pancreatic cancer cells than in normal pancreatic cells, and this phenomenon was also correlated with the transcription level of PD-L1. The higher expression of MLL1 in pancreatic cancer enhances PD-L1(CD274) expression by increasing H3K4me3 status at the CD274 promoter region, thereby helping pancreatic cancer escape anti-PD-L1/PD-1 immunotherapy [173].

Elevated MLL2 expression in esophageal squamous cell cancers (ESCC) and pancreatic ductal adenocarcinoma (PDAC) predicts poor prognosis in both tumors [174,175]. Additionally, a truncation mutation in MLL2 found in small cell lung cancer (SCLC) is known to interfere with the control of transcription enhancers, potentially contributing to the promotion of SCLC [176]. MLL3 is one of the most commonly mutated genes and has diverse functions in different cancers [110,127,162,177,178]. It has been suggested that MLL3 may function as a prognostic predictor of pancreatic cancer because it affects the cell cycle and DNA replication pathway in pancreatic ductal adenocarcinoma (PDAC) [174]. Similar to MLL1 in pancreatic cancer, overexpression of MLL3 in prostate cancer can also activate PD-L1 expression via regulation of the PD-L1 enhancer region, suggesting its potentially vital role in overcoming immune evasion in cancer cells [179]. In contrast, MLL3 is also considered a tumor suppressor that regulates DNA damage in bladder cancer [177].

SETD1A expression was upregulated in hepatocellular carcinoma compared with normal tissues, and its expression levels and sensitivity to sorafenib were negatively correlated. Functionally, it acts as an initiator to augment primary resistance to sorafenib by impairing the phosphorylation of Hippo signaling key factor YAP at serine 127 and enhancing YAP activation in hepatocellular carcinomas [180]. The pRB1-SETD1A complex formed by the action of CUDR in liver cancer cells labels H3K4me3 in the TRF2 promoter region, and ultimately, overexpressed TRF2 binds to the telomere repeat DNA to extend telomere length [181]. 

Upregulation of SETD1B was found to activate iNOS expression in tumor-induced myeloid-derived suppressor cells, which could provide a foundation for establishing an effective approach for improving the efficacy of cancer immunotherapy by targeting SETD1B [182]. SETD1B is also overexpressed in clear cell renal cell carcinoma (ccRCC), has been associated with cancer metastasis and has been reported as a promising prognostic indicator for ccRCC [183]. 

Recurrent mutations in SMYD1 have been identified in splenic marginal zone lymphoma using whole-exome sequencing (WES) and copy number variation analysis [184]. SMYD2 overexpression promotes disease progression and correlates with worse outcomes in other types of tumors, including esophageal squamous cell carcinoma [185], primary hepatocellular carcinoma (HCC) [186], and papillary thyroid carcinoma [187]. In non-small cell lung cancer (NSCLC), SMYD2 regulates the P53 signaling pathway to promote cisplatin resistance in NSCLC [188]. Notably, SMYD2 and miR-125b repression improved the sensitivity of multiple anticancer drugs by MDR 1 expression [189]. In contrast, low SMYD2 expression is associated with low survival in patients with renal cell carcinoma (RCC) [190] associated with complex karyotype acquisition and cancer progression [191]. 

SMYD3 is amplified in various tumor types. SMYD3 regulates the MEK/ERK kinase signaling pathway via methylation of mitogen-activated protein kinase 2 (MEKK2) in Ras-driven pancreatic ductal adenocarcinoma and lung adenocarcinoma cancers [192]. In ovarian cancer, SMYD3 regulates tumor proliferation and apoptosis by downregulating CDKN2A through tri-methylation of H4K20 and upregulating BIRC3 through tri-methylation of H3K4 [193]. SMYD3 was also reported to stimulate homologous recombination (HR)-related genes to modulate DNA repair in many cancer cell lines [194]. Most recently, overexpressed SMYD3 enhanced MMP2 and CDK2 expression in hepatocellular carcinoma and upregulated Bcl-2, Bcl-xl, MMP-2, and MMP-9 in NSCLC to facilitate tumorigenicity and cancer progression [195,196]. Under hypoxic conditions, SET7/9 has been shown to mediate the survival of various cancer cells against hypoxic stress by promoting HIF-1α protein stability and enhancing HIF-1-mediated gene transcription in osteosarcoma, hepatoma, and renal cell carcinoma [197]. In contrast, SET7/9 was shown to interact with, and methylate, β-catenin at lysine 180 to decrease β-catenin stability, which influences Wnt signaling and inhibits cervical cancer cell proliferation [198]. In that study, they showed that the depletion of SET7/9 in HeLa cells promoted the expression of Wnt/β-catenin target genes such as *c-myc* and *cyclin D1* and the growth of cancer cells. The mechanisms underlying the action of H3K4 HMTs on drug-resistant cancers are summarized in Table 1.

## 4. Inhibitors Targeting H3K4-Specific HMTs for Anticancer Therapy

### 4.1. WDR5 Inhibitors

Thus far, only a limited number of selective small-molecule compounds that can directly inhibit the active sites of enzymes of specific KMT2 family proteins have been reported [199]. Perhaps apart from the establishment of a high-efficiency screening method using the core SET domain, this is because the function of several subunits constituting the KMT2 complex contributes significantly to the overall H3K4 methylation activity. As mentioned above, H3K4 methyltransferase on its own has very weak methyl transfer catalytic activity, but binding of the RBBP5, WDR5, ASH2L, and DPY30 subunits leads to a significant increase in methyltransferase activity. Therefore, attempts have been made to inhibit the function of the MLL complex subunits. Among these methyltransferase adapter proteins, WDR5 is considered an attractive target because of its great importance within the MLL complex, which is responsible for its interaction with chromatin-remodeling proteins, transcription factors, and long non-coding RNAs. Additionally, by forming protein complexes with other proteins such as MYC, WDR5 induces the expression of key oncogenes, leading to tumor initiation, cell cycle progression, DNA replication, invasion, and cancer metastasis. Therefore, WDR5 inhibitors have great potential as anticancer drugs. Based on the protein interaction information, peptide inhibitors were initially reported, and later, structural analysis led to the development of peptidomimetic inhibitors that inhibit WDR5–MLL1 binding (Table 2). MM-102, MM-401, and MM-589, designed as inhibitors targeting the interaction of WDR5/MLL1, effectively inhibited histone H3K4 methyltransferase activity and reduced oncogenic gene transcription, inhibiting the growth and cell cycle of MLL-rearranged leukemia cells. MM-102 had a Ki < 1 nM in the WDR5 binding assay. MM-102 was also shown to inhibit MLL1 H3K4 methyltransferase activity (IC_50_ = 0.4 μM) and acute leukemia cell growth in a dose-dependent manner [200].

The high binding affinity of MM-401 to WDR5 (Ki < 1 nM) was detected by the label-free BioLayer Interferometry (BLI/OctetRED) assay and confirmed by competitive fluorescence polarization (FP) experiments. MM-401 interferes with the WDR5–MLL1 interaction (IC_50_ = 0.9 nM) and inhibits the activity of MLL1 (IC_50_ = 0.32 µM) in the in vitro HMT assay [201]. MM-589, another peptidomimetic WDR5 inhibitor (Ki <1 nM), has been shown to inhibit H3K4 HMT activity of MLL1 with an IC_50_ value of 12.7 nM and potently inhibits the growth of human leukemia cell lines containing MLL translocation at sub-micromolar concentrations, which is > 40 times superior to that of MM-401 [202].

At the same time, a nonpeptide small-molecule compound was also discovered that targets the bond between MLL and WDR5. WDR5-0103 was reported to specifically and effectively antagonize the interaction between WDR5 and the Win motif of MLL, which could further compromise MLL catalytic activity [203]. Structural and biophysical analyses showed that this antagonist binds to the WDR5 peptide-binding pocket with a Kd of 450 nM and inhibits the catalytic activity of the MLL core complex in vitro. At concentrations up to 100 μM, WDR5-0103 showed no inhibitory effect on the human H3K4 methyltransferase SETD7 or six other HMTs (G9a, EHMT1, SUV39H2, SETD8, PRMT3, and PRMT5). The specificity of this low-molecular weight compound for the MLL complex shows the potential for further development of WDR5-dependent enzyme inhibitors associated with MLL-rearranged leukemia or other cancers through inhibition of protein–protein interactions.

OICR-9429, which was designed through additional modifications of WDR5-0103 series compounds, showed high-affinity binding with WDR5 and occupied the MLL1-binding pocket with WDR5, resulting in disruption of the WDR5–MLL1 interaction. Grebien et al. have shown that OICR-9429 can decrease the viability of C/EBPα-mutated AML cells, supporting its potential anticancer effect on non-MLL-rearranged leukemia and solid tumors [204]. Zhang et al. screened a library of 592 FDA-approved drugs by measuring changes in the HTRF signal to identify compounds that inhibit the histone methylase activity of MLL1 methylation [205]. Piribedil, previously used to treat Parkinson’s disease, exerted extraordinary activity in inhibiting MLL methyltransferase activity (EC_50_ = 0.18 μM). An in vitro assay for histone methyltransferase/acetyltransferase activity showed that piribedil had little effect on the activity of MLL4 (IC_50_ > 100 μM) and less potent effects on EZH2, NSD2, PRMT4, p300, and SMYD3, suggesting that piribedil specifically targets MLL1 as opposed to other SET domain-containing enzymes and effectively inhibits MLL-rearranged AML proliferation by interfering with the MLL1–WDR5 interaction [205]. 

The Win motif, located on the N-flanking region of the SET domain, is required by all six MLL family members for binding to WDR5. A previous report illustrated that a six-residue Win motif peptidomimetic (Win6mer) that binds to WDR5 and selectively inhibits the SETD1A and MLL1 complex was previously designed [206].

**Table 2:** Potential therapeutic inhibitors targeting H3K4-specific HMTs.

Inhibitor	Structure	Mode of Action	Kd or Ki	Methylation IC_50_	PPI IC_50_	GI_50_	Cancer Cell Type	In Vivo Validation	Ref
MM-102	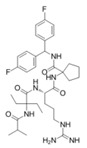	WDR5–MLL	<1 nM	400 nM	2.4 nM	25 μM	MLL1-rearranged leukemia		[200]
MM-401	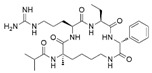	WDR5–MLL	<1 nM	320 nM	0.9 nM	5.9~12.6 μM	MLL1-rearranged leukemia		[201]
MM-589	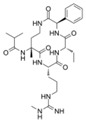	WDR5–MLL	<1 nM	12.7 nM	0.9 nM	0.21~0.25 μM	MLL1-rearranged leukemia		[202]
WDR5-0103	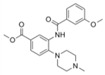	WDR5–MLL	450 nM	39 μM					[203]
OICR-9429	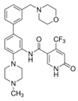	WDR5–MLL	30 nM			~5 μM	C/EBPα-mutant AML Ovarian cancer (with topotecan) Prostate cancer (with cisplatin)	Yes Yes	[204,207] [208] [209]
Piribedil	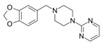	WDR5–MLL		180 nM		65~92 μM	MLL1-rearranged AML	Yes	[205]
Win6mer	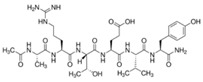	WDR5–MLLWDR5–SETD1A	2.9 nM	2.2 nM2.5 nM					[206]
Compound C6	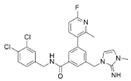	WDR5–MLL	0.1 nM	20 nM		2.5~6.4 μM	MLL1-rearranged leukemia		[210]
Compound 16	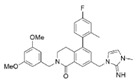	WDR5–MLL	<0.02 nM	2.2 nM		38~78 nM 0.26~0.49 μM	MLL1-rearranged leukemia Neuroblastoma and Burkitt’s lymphoma		[211]
MCP-1	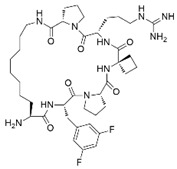	Menin–MLL	4.7 nM		18.5 nM		MLL1-rearranged leukemia		[212]
MI-2	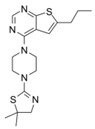	Menin–MLL	158 nM		446 nM	7.2~18 μM	MLL1-rearranged leukemia		[213]
MI-2-2	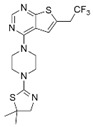	Menin–MLL	22 nM		46 nM	3 μM	MLL1-rearranged leukemia (with DOT1L inhibitor)	Yes	[214,215]
MI-463	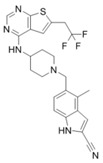	Menin–MLL	9.9 nM		15.3 nM	0.23 μM	MLL1-rearranged leukemia	Yes	[216]
MI-503	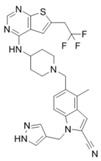	Menin–MLL	9.3 nM		14.7 nM	0.22 μM 1.5~11.7 μM	MLL1-rearranged leukemia Castration-resistant prostate cancer	Yes Yes	[216] [135]
MI-538	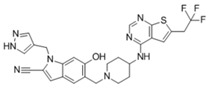	Menin–MLL	6.5 nM		21 nM	83 nM	MLL1-rearranged leukemia	Yes	[217]
MI-3454	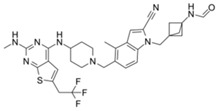	Menin–MLL			0.51 nM	7.6~27.1 nM	MLL1-rearranged or NPM1-mutated leukemia	Yes	[218]
BAY-155	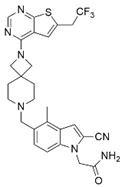	Menin–MLL	75 nM		8 nM	90~140 nM	MLL1-rearranged leukemia	Yes	[219]
VTP50469	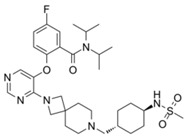	Menin–MLL	104 pM			13~37 nM	MLL1-rearranged leukemia	Yes	[220]
M-89	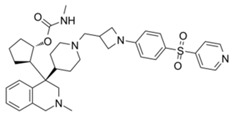	Menin–MLL	1.4 nM		5 nM	25~55 nM	MLL1-rearranged leukemia		[221]
M-525	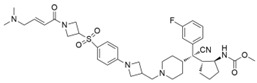	Menin–MLL			3.3 nM	2.3~10.3 nM	The first irreversible Menin inhibitor		[222]
M-808	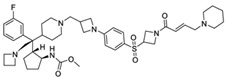	Menin–MLL			2.6 nM	1~4 nM	MLL1-rearranged leukemia	Yes	[223]
AZ505	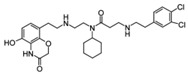	SMYD2	500 nM	120 nM			MDR-clear cell renal cell carcinoma (ccRCC) Triple-negative breast cancer	Yes	[224] [117]
LLY-507	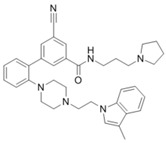	SMYD2		<15 nM		1.5~6 μM 1.77~2.9 μM	Esophageal, liver, and breast cancer cells High-grade serous ovarian carcinomas (HGSOCs).		[225] [226]
A-893.	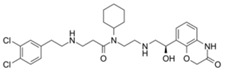	SMYD2		2.8 nM			Lung cancer		[227]
BAY598	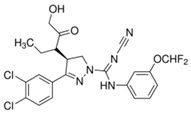	SMYD2	1.1~1.2 nM	27 nM		<10 μM	Esophageal cancer (combi w/doxorubicin)	Yes	[228]
EPZ031686	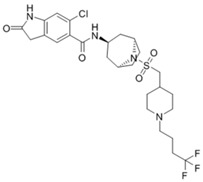	SMYD3	1.3~4.7 nM	3 nM					[229]
EPZ028862	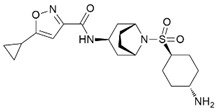	SMYD3		1.8 nM		>40 μM	Esophageal squamous cell carcinoma		[230]
GSK2807	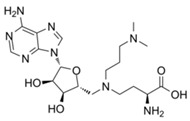	SMYD3	14 nM	130 nM					[231]
Compound 29	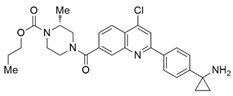	SMYD3	440 nM	11.7 nM		17.7 μM (2D) 1.04 μM (3D)	Hepatocarcinoma		[232]
*(R)*-PFI-2	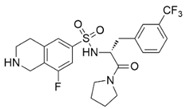	SETD7/9	0.33 nM	2 nM			Breast cancer cells		[233]

The six-residue peptidomimetic Win6mer, designed selectively in the MLL1 and SETD1A core complex, destroys the Win motif–WDR5 interface required to stabilize contact with the RBBP5/ASH2L heterodimer. Win6mer binds to WDR5 with a Kd of 2.9 nM. 

Recently, Aho et al. identified compound C6, which inhibits WDR5–MLL1 binding with picomolar binding affinity through fragment-based screening of 13,800 compounds and structure optimization [210]. Additionally, further optimization of compound C6 led to the generation of compound 16, which contained a stronger dihydrothioquinolinone structure [211]. This compound also has a picomolar binding affinity and inhibits the proliferation of MYC-induced cancer cells (neuroblastoma and Burkitt’s lymphoma) and MLL1-rearranged leukemia by reducing MYC recruitment to the MYC/WDR5 target gene.

### 4.2. Menin Inhibitors

The protein menin binds to the N-terminal fragment of MLL retained in all MLL fusion proteins, which plays a critical role as an oncogenic cofactor of MLL fusion proteins in leukemia [147]. Thus, inhibiting the interaction between menin and histone methyltransferases could be a novel therapeutic strategy. Initially, macrocyclic peptidomimetic inhibitors (MCP-1) were designed to inhibit the menin−MLL1 interaction. The most potent MCP-1 binds to menin with a Ki value of 4.7 nM and is >600 times more potent than the corresponding acyclic peptide. However, the disadvantage is that cell permeability must be secured to maintain the in vivo activity of MCP-1. Optimization of MCP-1 may ultimately yield a class of potent and cell-permeable small-molecule inhibitors of the menin−MLL1 interaction as potential new therapeutics to treat acute leukemia with MLL1 rearrangements [212]. 

MI-2-2 is a thienopyridine-based compound optimized from MI-2 [214,215]. Despite its excellent ability to inhibit the binding of menin–MLL1, its in vivo activity is limited due to its poor metabolic stability. However, it has inspired the development of a variety of low-molecular-weight compounds with improved activity. MI-463 and MI-503 directly bind to menin with low nanomolar binding affinities and effectively block the menin–MLL interaction [216]. Pharmacological inhibition of the menin–MLL interaction using small molecules blocks the progression of MLL leukemia in vivo without impairing normal hematopoiesis. These compounds can reach the target protein in mammalian cells and effectively inhibit the menin–MLL–AF9 interaction at sub-micromolar concentrations, as assessed in the co-immunoprecipitation experiment. Treating murine bone marrow cells (BMC) transformed with the MLL-AF9 oncogene with MI-463 or MI-503 resulted in significant growth inhibition, with half-maximal growth inhibitory concentration (GI_50_) values of 0.23 μM and 0.22 μM, respectively. Both compounds have an appropriate pharmacokinetic profile and high oral bioavailability, and their in vivo administration results in a substantial extension of survival in a mouse model of MLL leukemia through the on-target mechanism of action. Importantly, these compounds do not impair normal hematopoiesis in mice, providing evidence that a sufficient therapeutic window can be achieved [216]. The effectiveness of MI-505 has been proved, not only in leukemia, but also in castration-resistant prostate cancer [135]. MI-503 induces apoptosis by effectively inhibiting the interaction between menin and MLL in VCaP and LNCaP cells, thereby reducing the expression of PSA protein and AR-target genes. Additionally, treatment with MI-503 in the LNCaP-AR xenograft model and the VCaP xenograft mouse castration model showed marked tumor growth inhibition. These findings suggest that inhibiting the menin–MLL1 interaction using MI-503 may be a novel treatment for hormone-refractory prostate cancer. 

BAY-155 was recently reported to be an improved menin inhibitor [219]. BAY-155 has an inhibitory IC_50_ of 8 nM in a time-resolved fluorescence resonance energy transfer (TR-FRET) assay to interfere with menin–MLL binding, which more strongly interferes with menin–MLL binding compared to MI-503. Isothermal titration calorimetry (ITC) experiments confirmed that BAY-155 binds menin protein in a 1:1 ratio, with a binding affinity of 75 nM. Additionally, BAY-155 showed a significantly improved selectivity profile compared to that of MI-503. In an assay panel covering 77 pharmacologically relevant safety targets, including G-protein coupled receptors (GPCRs), ion channels, and transporters, 10 µM BAY-155 inhibited only seven tested proteins, while MI-503 inhibited 28 proteins at the same concentration [219]. 

In most MLL-rearranged leukemia models, the response to DOT1L inhibitors is limited. One of the reasons for this is the role of the wild-type MLL complex and fusion-MLL protein in leukemogenesis. Accordingly, a combination of a DOT1L inhibitor (EPZ004777) and an MLL–menin interaction inhibitor (MI-2-2) was administered and showed dramatically improved differentiation induction and apoptosis in various MLL disease models, including primary leukemia cells, suggesting its potential as a new strategy for the treatment of leukemia in the future [214].

Unlike most WRD5 inhibitors, which exhibit moderate activity in most cells, hence limiting further application of the compound in animal tumor models, there is potential for the development of potent and selective menin inhibitors. MI-3454 is a newly designed structural analog that utilizes the crystal structures of MI-503 and menin [218]. It blocks the interaction between menin and MLL1 at subnanomolar concentrations, resulting in almost 60 times greater improvement over MI-503. Additionally, MI-3454 shows superior activity across MLL leukemia cells compared with all menin–MLL1 inhibitors reported to date. MI-3454 induced complete remission or regression of leukemia in a mouse xenograft model of MLL1 translocation or NPM1 mutant leukemia derived from AML patients. Most recently, KO-539, a structurally related analog of MI-3454, has been approved for clinical trials (Table 3), further amplifying interest in this field. KO-539 is expected to reduce the transcription of HOXA9 and MEIS1 promoters and lead to terminal differentiation of AML blasts following oral administration once daily in patients with MLL1-rearranged or NPM1-mutant AML cells. Other potent and selective menin–MLL inhibitors, JNJ-75276617 and SNDX-5613 (VTP-50469), have entered Phase 1 and Phase I/II clinical trials, respectively, for relapsed/refractory acute leukemia.

### 4.3. SMYD Inhibitors

SMYD2, which has less limited substrate specificity, is attracting attention as an anticancer target molecule because of the negative correlation between expression levels and patient survival rates in various cancer types. SMYD2-specific inhibitor, AZ505, was identified in high-throughput screening, showing an IC_50_ of 120 nM in an enzyme assay as well as high selectivity indices (>690-fold) against other methyltransferases [224]. LLY-507, another potent small-molecule inhibitor of SMYD2, showed >100-fold selectivity over a broad range of methyltransferase and non-methyltransferase proteins. The ability of LLY-507 to inhibit the catalytic function of SMYD2 was tested by a scintillation proximity assay (SPA) using peptides derived from a previously described p53, an SMYD2 substrate. LLY-507 potently inhibited the ability of SMYD2 to methylate the p53 peptide with an IC_50_<15 nM. In the cell-based assay, LLY-507 also inhibited monomethylation of the Lys 370 residue of the p53 protein mediated by SMYD2 at sub-micromolar concentrations. However, cellular global histone methylation levels were not significantly affected by the inhibition of SMYD2 with LLY-507. Subcellular fractionation studies indicated that SMYD2 was primarily localized in the cytoplasm, suggesting that SMYD2 targets a very small subset of histones at specific chromatin loci and/or non-histone substrates [225]. 

Additionally, A-893 is a benzoxazinone-based inhibitor that exhibited a high level of selectivity for SMYD2 in an inhibition assay using 30 methyltransferase panels [227]. An analysis of the cocrystal structure of A-893 highlights the contribution of the newly installed hydroxyl group to an intricate network of hydrogen bonds around the lysine pocket of SMYD. This compound inhibited SMYD2 with an IC_50_ of 2.8 nM in the SPA assay measuring the methylation of p53 peptide by SMYD2. Furthermore, in A549 cells, A-893 reduced the monomethylation of p53 K370 by SMYD2. However, the cellular activities of these three SMYD2 inhibitors are limited. Eggert et al. designed a potent and selective amino-pyrazoline-based small-molecule SMYD2 inhibitor [228]. BAY-598 inhibited SMYD2 with an IC_50_ of 27 ± 7 nM in a biochemical SPA assay and an IC_50_ of 58 nM in a cell-based assay measuring the methylation activity of p53. In the same study, oral administration (100 mg/kg, q.d.) of BAY-598 significantly reduced SMYD2-dependent methylation signals in an esophageal ex vivo model. However, BAY-598 alone failed to show an anti-proliferative effect in cell-based assays and in vivo xenograft models. BAY-598 (500 mg/kg, q.d.) significantly reduced tumor growth in an in vivo esophageal xenograft model only combined with doxorubicin, suggesting the potential for limitations in the application of SMYD2 inhibitors to specific anticancer drugs in response to certain types of cancer [228].

SMYD3 is overexpressed in different types of tumors, including breast, gastric, pancreatic, colorectal, lung, and hepatocellular carcinoma [121,192,234,235]. EPZ031686, the first orally available SMYD3 inhibitor, was identified by screening the Epizyme proprietary histone methyltransferase-biased library. This compound inhibited SMYD3 with an IC_50_ of 3 nM in the SPA assay, which measured the methylation of MEKK2 by SMYD3 [229]. EPZ031686 competitively inhibits SAM with Ki = 4.7 nM and non-competitively inhibits MEKK2 with a 1.3 affinity. After oral administration of 50 mg/kg of EPZ031686 to mice, plasma concentration remained above IC_50_ for more than 12 h, highlighting its possible efficacy in future in vivo models [229]. EPZ030456 could not be tested in vivo because of its low solubility [229]. Structure–activity relationship studies of SMYD3 inhibitors have led to identification of the isoxazole sulfonamide series exemplified by EPZ028862 [230] and this compound inhibited SMYD3 with a biochemical IC_50_ of 1.80 ± 0.06 nM in the SPA assay. Its mechanism of action is mixed-type inhibition of SAM and non-competitive inhibition of MAP3K2. Moreover, EPZ028862 showed selectivity for SMYD3 in an assay using a panel of 16 methyltransferase enzymes [230]. GSK2807 (Ki = 14 nM), a potent and selective SAM competition inhibitor, inhibits the activity of SMYD3 by forming a dead-end ternary complex of SMYD3, GSK2807, and MEKK2 [231]. BAY-6035 is a selective and substrate-competitive SMYD3 inhibitor which was discovered through scientific collaboration between the Structural Genomics Consortium (SCG) and Bayer AG (https://www.thesgc.org/chemical-probes/BAY-6035 (accessed on 24 June 2021)), although the corresponding publication has not been published thus far. BAY-6035 showed nanomolar IC_50_ values in vitro and in cell-based assays (88 and 70 nM, respectively) for the methylation of MEKK2. Compound 29, a novel class of tetrahydroacridine compounds, exhibited high potency by irreversibly inhibiting SMYD3 enzymatic activity (IC_50_ = 0.0117 ± 0.0109 μM in the SPA assay) and showed antiproliferative activity against HepG2 cells in 3D cell culture (GI_50_ = 1.04 μM [232]). 

### 4.4. Other Inhibitors 

SET7/9 has been shown to have a very broad target specificity in vitro, including transcriptional regulators such as TAF10, p53, ER, p65, STAT3, Rb, MYPT, Tat, and FOXO3. Based on its properties, SET7/9 may be involved in various molecular pathways related to metabolism, inflammation, and cancer. (R)-PFI-2 is a first-in-class, potent (Ki *app* = 0.33 nM), selective, and cell-active inhibitor of SET7/9 with an IC_50_ value of 2.0 ± 0.2 nM [233]. (R)-PFI-2 inhibits SETD7 activity as a cofactor-dependent substrate competition inhibitory mechanism by occupying the substrate peptide-binding groove of SETD7 and direct contact with the donor methyl group of the cofactor. The binding of (R) -PFI-2 to endogenous SETD7 has also been confirmed in MCF7 cells, and the role of SETD7 in the signaling of the Hippo pathway that controls cell growth and organ size has been elucidated. Although anticancer studies using this compound have not been reported thus far, it provides a useful tool that can be used in future studies to confirm the roles of SETD7 and its potential as a target protein for drug development.

PRDM9 is a PR domain-containing protein that trimethylates histone 3 on lysine 4 and 36. PRDM9 is involved in the expression of meiosis-specific cancer/testis genes [236]. There is increasing evidence that PRDM9 may be involved in oncogenesis and/or cancer evolution [237]. MRK-740 is a potent (IC_50_ = 80 ± 16 nM) selective PRDM9 inhibitor, and its activity has also been demonstrated in cell-based assays. It binds to the substrate-binding pocket and acts as a SAM-dependent substrate competition inhibitor with an abnormally broad interaction with the cofactor S-adenosylmethionine (SAM). In cells, MRK-740 specifically and directly inhibits H3K4 methylation at endogenous PRDM9 target loci [238]. However, MRK-740 treatment does not affect the proliferation of multiple myeloma lines and breast cancer cell lines, and its usefulness as a target molecule in cancer is still uncertain.

### 4.5. Protein Degraders

Protein silencing has often been used to investigate protein function. Several types of protein silencing methodologies have been developed for therapeutic purposes, including targeting coding genes, transcripts, and translation processes. Direct degradation of the target protein can overcome some of the limitations of previously described methods. The first cases of PROTAC compounds targeting BET family proteins were reported by several groups in 2015 [239,240,241], and a large number of small-molecule proteolytic agents are currently under development. 

Although slow compared with other fields, the development of PROTAC, which regulates the function of H3K4 methyltransferase, is also drawing attention. One of the important subunits of the KMT2 complex, WDR5, has a WD-repeat domain that mediates protein interactions. The primary aim of the researcher is to design PROTAC to induce selective degradation of WDR5 using OICR-9429, a low-molecular substance capable of pharmacologically targeting WDR5, hence confirming whether the proliferation of cancer cells is inhibited by destroying the KMT2 complex in cancer cells. Although specific PROTAC design and intracellular activity have not been reported thus far, interesting results are expected in the future, considering the role of WDR5 in H3K4 methyltransferase activity.

## 5. Conclusions

Anticancer drug resistance is one of the major challenges that needs to be solved, although the mechanisms involved are very complex. Extensive evidence generated over the past decade shows that H3K4 methyltransferases and demethylases mediate the actions of several genes associated with drug resistance. Therefore, targeting H3K4 methyltransferase is expected to exert a synergistic effect, either on its own or in combination with other drugs, to overcome drug resistance in leukemia and various solid cancers. The majority of research supports that H3K4 methyltransferases promote drug resistance, although there is some evidence suggesting their suppressive effect in developing cancers. H3K4 methyltransferase function generally requires enzyme activity. However, non-enzymatic roles have also been reported. Additionally, several members of the H3K4 methyltransferase family have been well identified. Nevertheless, studies on the detailed mechanisms of action of these proteins are limited, and further exploration is required. Importantly, limited inhibitors specifically targeting H3K4 methyltransferase may be clinically available in the near future. However, efforts to design or optimize clinically potent H3K4 methyltransferase inhibitors targeting enzyme activity, binding activity with other factors, or protein stability, must continue. Finally, we need to generate conclusive evidence of the contribution of HMT to drug resistance. These efforts, combined with current treatments for drug-resistant cancers, could provide more treatment options.

## Figures and Tables

**Table 1:** Potential role of H3K4 HMTs in drug-resistant cancers.

Enzyme	Cancer Types	Proposed Mechanism
MLL1	Chemotherapy resistant MLL leukemia Castration-resistant prostate cancer Anti-PD-L1/PD-1 resistant pancreatic cancer	Increases MDR-1 expression [170] Activates androgen receptor signaling [135] Increases PD-L1 expression [173]
MLL3	Tamoxifen-resistant breast cancer Anti-PD-L1/PD-1 resistant prostate cancer	Increases ERα expression [111] Increases PD-l expression [179]
SETD1A	Tamoxifen-resistant breast cancer Tamoxifen-resistant breast cancer Triple-negative breast cancer Castration-resistant prostate cancer Sorafenib resistant hepatocarcinoma	Increases ERα expression [111] Activates ERα signaling and EGFR expression [119] Activates MMP expression [113] Activates FOXM1 signaling via binding with E2F1 [143] Activates Yes-associate protein [180]
SETD1B	Triple-negative breast cancer	Regulates adiponectin receptor 1 signaling [116]
SMYD2	Triple-negative breast cancer Oxaliplatin-resistant colon cancer Cisplatin-resistant non-small cell lung cancer Chemotherapy resistant renal cell carcinoma	Activates STAT3 and the p65 [117] Increases MDR-1 expression [134] Inhibits p53 signaling [188] Increases MDR-1 expression [189]
SMYD3	Cisplatin-resistant breast cancer	Increases WNT10B expression and promote the EMT [120,121]
SET7/9	Anti-estrogen-resistant breast cancer Castration-resistant prostate cancer	Controls the stability of E2F1 and DNMT1 [126] Alters chromatin accessibility via frameshift mutation [142]

**Table 3:** Inhibitors of H3K4-specific HMTs in a clinical trial.

Drug Name	Status	Mechanism	Cancer Type	Administration	ClinicalTrial.gov ID#
KO-539	Phase 1/2a	Menin–MLL1 inhibitor	Relapsed or Refractory Acute Myeloid Leukemia	Oral	NCT04067336
JNJ-75276617	Phase 1	Menin–MLL1 inhibitor	Acute Leukemias Acute Myeloid Leukemia Acute Lymphoblastic Leukemia	Oral	NCT04811560
SNDX-5613	Phase 1/2	Menin–MLL1 inhibitor	Acute lymphoblastic leukemia (ALL) Mixed phenotype acute leukemia (MPAL). Acute Myeloid Leukemia (AML). NPM1c AML.	Oral	NCT04065399

## Data Availability

Not applicable.
